# Experimental and Numerical Study of the Laminar Burning Velocity and Pollutant Emissions of the Mixture Gas of Methane and Carbon Dioxide

**DOI:** 10.3390/ijerph19042078

**Published:** 2022-02-12

**Authors:** Yalin Wang, Yu Wang, Xueqian Zhang, Guoping Zhou, Beibei Yan, Rob J. M. Bastiaans

**Affiliations:** 1 School of Environmental Science and Engineering, Tianjin University, Tianjin 300072, China; yanbeibei@tju.edu.cn; 2 Departments of Mechanical Engineering, Eindhoven University of Technology, P.O. Box 513, 5600 MB Eindhoven, The Netherlands; y.wang14@tue.nl (Y.W.); r.j.m.bastiaans@tue.nl (R.J.M.B.); 3 Jinan Energy Investment & Holding Group Co., Ltd., Jinan 250013, China; dreampolice@163.com; 4 EBICO (China) Environment Co., Ltd., Wuxi 214125, China; zgp@ebico.com; 5 Key Laboratory of Efficient Utilization of Low and Medium Grade Energy, Ministry of Education, School of Mechanical Engineering, Tianjin University, Tianjin 300072, China

**Keywords:** laminar burning velocity measurement, pollutant emissions in premixed combustion, one-dimensional flame simulation

## Abstract

This paper presents the experimental and numerical study of the laminar burning velocity and pollutant emissions of the mixture gas of methane and carbon dioxide. Compared to previous research, a wider range of experimental conditions was realized in this paper: CO_2_ dilution level up to 60% (volume fraction) and equivalence ratio of 0.7–1.3. The burning velocities were measured using the heat flux method. The CO and NO emissions after premixed combustion were measured by a gas analyzer placed 20 cm downstream of the flame. The one-dimensional free flames were simulated using the in-house laminar flame code CHEM1D. Four chemical kinetic mechanisms, GRI-Mech 3.0, San Diego, Konnov, and USC Mech II were used in Chem1D. The results showed that, for laminar burning velocity, the simulation results are all lower than the experimental results. GRI Mech 3.0 showed the best agreement when the CO_2_ content was below 20%. USC Mech II showed the best consistency when the CO_2_ content was between 40 and 60%. For CO emission, these four mechanisms all showed a small error compared with the experiments. When CO_2_ content is higher than 40%, the deviation between simulation and experiment becomes bigger. When the CO_2_ ratio is more than 20%, the proportion of CO_2_ does not affect CO emission so much. For NO emission, when the CO_2_ content is 40%, the results from simulation and experiment showed a good agreement. As the proportion of CO_2_ increases, the difference in NO emissions decreases.

## 1. Introduction

Biogas is one of the renewable fuels that is produced in many different sources, such as sewage sludge, landfills, and organic material [1]. Methane (CH_4_) is the main component of biogas. It is valuable but also harmful to the environment. Biogas can be used for heat, electricity, vehicles, etc., to reduce environmental emissions and the use of fossil fuels. CH_4_ and carbon dioxide (CO_2_) are the two main components in biogas. CH_4_ accounts for 55 to 65% (volume fraction) and CO_2_ accounts for 35 to 45% in the biogas from sewage digesters. CH_4_ accounts for 60 to 70% and CO_2_ accounts for 30 to 40% of the biogas from organic waste. CH_4_ accounts for 45 to 55% and CO_2_ accounts for 30 to 40% in the biogas from landfills [2].

The usage of biogas will become more and more widely in the future. It is important to research its experimental characterization of fundamental combustion parameters [3]. Among a variety of combustion parameters, the laminar burning velocity (S_L_) is an important requisite to assess flame quenching, flashback, blow-off, and stabilization [4]. The laminar burning velocity is defined as the speed at which an unstretched, adiabatic, and premixed planar flame propagates relative to the unburned mixture [5]. It is a property of fuel–oxidizer mixtures and is a function of initial temperature (T), pressure (P), and equivalence ratio (Φ). Experimental techniques like the counterflow flame method [6], the heat flux method (HFM) [7], and the spherical flame method [8] can be applied in determining this property. To get the S_L_, the one-dimensional and adiabatic flame has to be obtained. For the experiments in this study, the authors use HFM, which was proposed by de Goey et al. [7]. This method stabilizes a stretch less one-dimensional flame without net heat flux and is capable of determining accurate adiabatic laminar burning velocity.

The laminar burning velocity of biogas can also be found in the literature. Cohé et al. [9] used a Bunsen burner to measure the S_L_ of the mixture of CH_4_ and CO_2_. They got the conclusion that the pressure affected the S_L_ much more than the dilution of CO_2_. Additionally, they used PREMIX code from the program package CHEMKIN-II to simulate biogas under an equivalence ratio of 0.88, and 0.1 and 0.2 MPa pressure, 300 K temperature, then they approved that the percentage reduction in S_L_ corresponded to the percentage of CO_2_. The chemical kinetics was GRI-Mech v3.0. Experimentally, Galmiche et al. [10] and Halter et al. [11] used a cylindrical vessel to measure the S_L_ from an initial pressure of 0.1 MPa and an initial temperature of 393 K but with up to 20% CO_2_ in a stoichiometric mixture with methane linearly, but the decrease of the laminar burning velocity does not show a linear trend. Xie et al. [12] used a constant volume chamber to study the flame instability and flame radiation of CH_4_/CO_2_/O_2_ mixtures. Chen et al. [13] carried out research on the effects of diluents on laminar flame speeds of stoichiometric, laminar, and premixed dimethyl ether (DME)/air flames. Qiao et al. [14] used a spherical vessel to measure the S_L_ of premixed methane/air/diluent flames. However, it is difficult to extract the S_L_ from associated conical flames because of curvature effects at the tip and the base. Therefore, de Goey and his colleagues [15] introduced HFM for measuring adiabatic burning velocity and stabilizing flat flames canceling heat fluxes. In recent times, Hermanns [16] used HFM to determine the effect of H_2_ addition to the S_L_ of methane–air mixtures. Konnov and his colleagues [17,18,19,20] used a replica of the HFM setup and measured the S_L_ of CH_4_, C_2_H_6,_ and CH_4_-H_2_ mixtures with different dilution ratios of artificial air having CO_2_, N_2,_ or Ar. In contrast again, Clarke et al. [21] used the constant volume technique at zero gravity conditions for the determination of burning velocities of natural gas-like compositions (i.e., methane/diluents–air mixtures). But the stretch corrected data of methane-diluent mixtures do not appear to be available in the literature.

The present work is an experimental and computational investigation into the effect of dilution with CO_2_ on the adiabatic burning velocity partially along the lines of Nonaka and Pereira [3] but with extended CO_2_ amounts up to 60%, and additionally CO and NO emission. As NO and CO are very harmful to the human body, NO_x_ is a concern because they contribute to the formation of acid rain, which threatens human health [22,23]. Furthermore, for simulating, we investigate the role of diffusion approximations. The experimental method is HFM. The setup is validated initially by obtaining S_L_ values from the measurements of ethane-air mixtures and methane–air mixtures, and also made many comparisons with the literature. We made a closed chamber outside the burner, and installed a probe inside the chamber to measure the NO and CO emissions. The computational method is performed by a steady one-dimensional laminar flame code, Chem1D [24]. The reaction mechanisms are four different mechanisms which are GRI-Mech 3.0 [25], San Diego [26], Konnov [27], and USC Mech II [28], a suitable selection also used by Nonaka and Pereira [3], to predict the S_L_ of the mixtures in and to compare the results from the experiment.

## 2. Materials and Method

There are two main methods, stationary flame and non-stationary flame methods [29], to obtain the laminar burning velocity. Stationary flames can be established using a burner in which the premixed fuel and oxidant are provided at constant mass flow. Then the flame will establish at the outlet of the burner. Having small experimental deviations, flat flame burners provide the closest approximation to the unstretched flat 1-D flame. Flat flame burners were first used by Powling [30] and then developed by Bosschaart and de Goey [31], amongst others. They reduced the problem of heat transfer and allowed adiabatic burning velocities to be obtained without corrections.

### 2.1. Heat Flux Burner

**Figure 1:**
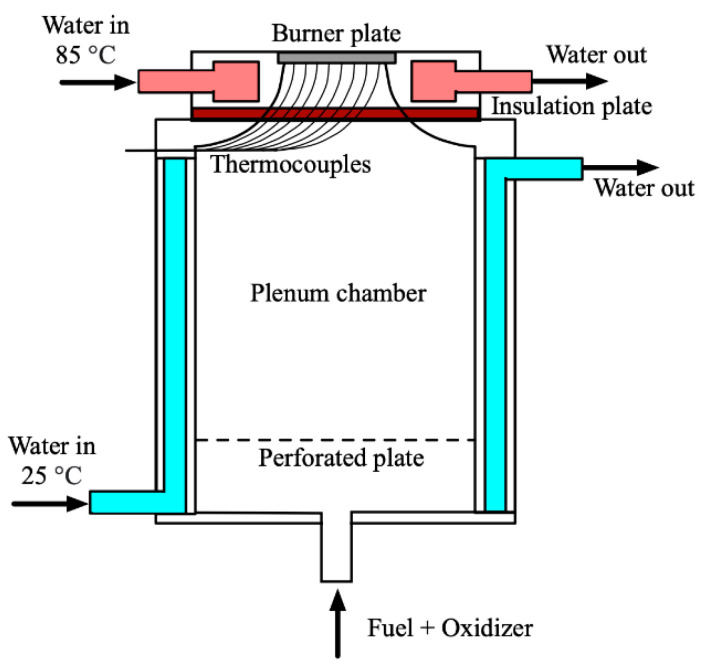
Schematic diagram of the heat flux burner.

As already indicated, the HFM is applied for the determination of S_L_ in this paper. Figure 1 shows the heat flux method burner (HFB). There is a burner head on the top and a plenum chamber on the bottom. From the chamber, a uniform flow will be formed. The burner plate is 1 mm thick. The inlet for the premixed fuel–oxidizer mixture is placed on the bottom of the plenum chamber. The cooling jacket is around the chamber with an inlet water temperature of 25 °C to keep the inlet gases temperature at a constant level. The heating jacket is located around the burner plate. The inlet water temperature is 85 °C, to keep a stable suitable temperature of the burner plate, having an exothermal flame on top of it, with zero net heat flux by adjusting the mass flow. There are several thermocouples attached to the burner plate to measure the radial temperature of the burner plate.

### 2.2. The Principle of the Experiment

**Figure 2:**
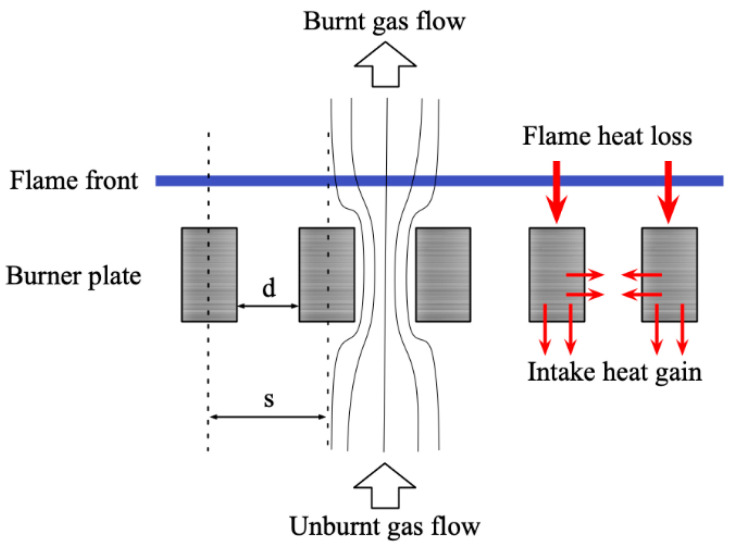
The heat flux compensation mechanism for the one-dimensional adiabatic laminar flame.

In HFB’s, there is no flame stretch and flow straining. This adiabatic flame condition can be realized by adding the heat from the burner plate to the unburnt gas portion to make up the heat loss from the flame to the burner head [32]. The mechanism is shown in Figure 2. The temperature distribution of the burner plate is as the following equation:

(1) $$T_{p\left(r\right)}=T_{center}+Cr^{2},$$

in which  r represents radius from the center of the burner plate, $T_{p\left(r\right)}$ represents the temperature at different r, $T_{center}$ represents the temperature at the center of the burner plate and C represents a parabolic coefficient variant related to the heat transfer [7,31]. In the complete burner plate, there are several thermocouples placed at different r. These thermocouples would show the data of $T_{p\left(r\right)}$ and C. When the value of C is zero, the temperature distribution is flat, then the adiabatic flat flame is obtained. The diameter of each hole is indicated by d and s is the center distance between each hole.

### 2.3. Experimental Design

**Figure 3:**
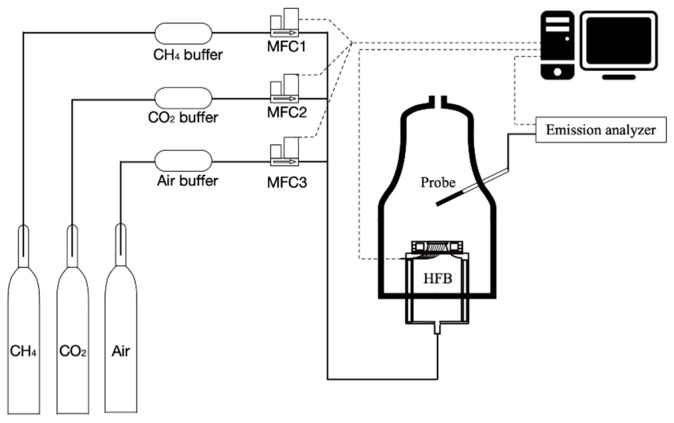
Schematic design of the setup.

**Figure 4:**
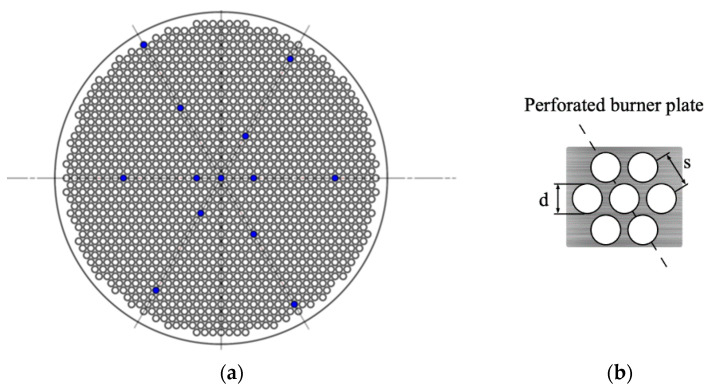
Burner plate. (**a**) The perforated burner plate with 13 thermocouples (blue dots) installed on it, (**b**) the holediameter and the pitchdistance.

Figure 3 shows the schematic design of the setup used in this study. This HFM setup was built for gaseous fuels at atmospheric pressure. The current burner plate is shown in Figure 4, as follows. The perforation pattern was 0.4 mm holediameter and 0.51 mm pitchdistance. The outer diameter is 20 mm; there were 1362 holes totally in this burner plate. The material of the burner plate was brass and the material of the insulation plate was nylon. Several thermocouples were placed flush-mounted in the plate. There was a computer connected to all thermocouples to run Labview for analyzing the data. The types of mass flow controller (MFC) are Bronkhorst FG-201CV-RAD-33-V-DA-000 for CO_2_, FG-201CV-RAD-33-V-DA-A1V for CH_4_ and air. The range of pressure for all MFCs were from 0–5 bar. All MFCs were calibrated with professional equipment and set up before the experiment. The left part in Figure 3 is the gas supply part. The purity of CO_2_ is 99.995%. The purity of CH_4_ is 99.5%. The experimental conditions are 1 atm and 298 K. A gas analyzing probe is located 20 cm above the HFB. The series of the gas analyzer is MRU VARIO Plus. It can give real-time and long-term monitoring for several emission gases, such as CO, CO_2_, CH_4_, NO, NO_2_, SO_2_, H_2_S, H_2_, etc. This is a portable stack gas emission analyzer for long time measurements of industrial combustion. The outstanding benefits of it are that it is the most suitable method for low NO_x_ measurements and other toxic gas emissions measurements. The accuracy of it is 5% for NO and 3% for CO.

### 2.4. Error Analysis

Alekseev et al. [33] identified the uncertainties in laminar burning velocity in the HFM setup and the HFB method in the experiment. The main reason for uncertainties is the uncertain value of the parabolic coefficient C. The factors include the temperature of the burner plate, the mass flow, the control from the MFC, and the inlet gas temperature. The value of C can be obtained from linear regression of the temperature data over the burner plate. The uncertainty $\sigma_{C}$ can be shown as the following equation:

(2) (1N−2∑i(Ti−C·(r2)i−Tcenter)2∑i((r2)i−(r2¯))2)12

In Equation (2), N represents the number of thermocouples, $T_{i}$ is the thermocouple reading at the distance of r and $\left(\bar{r^{2}}\right)$ is the mean of the squared r.

### 2.5. One-Dimensional Flame Simulation

The one-dimensional free flames were simulated using the in-house laminar flame code CHEM1D [24] to get solutions, including S_L_. CHEM1D solves a set of equations describing the conservation of mass, momentum, energy, and species for chemically reacting flow using an exponential finite-volume discretization in space [4]. Non-linear differential equations were solved with a fully implicit, modified Newton method along with an option to invoke several transport models, including the complex one. An adaptive gridding procedure was also achieved to increase accuracy in the flame front by placing almost 80% of the grid points in the area with the largest gradients [16]. Basic thermodynamic data were from Burcat and Ruscic [34]. CHEM1D can calculate not only with a simple one-step reaction mechanism but can also run with complex chemistry. Many kinetic mechanisms have been developed, such as Li et al. [35], Burke et al. [36], Kéromnès et al. [37], and GRI Mech 3.0 [25]. The users can use different mechanisms in Chem1D directly without editing. GRI-Mech 3.0 is an updated mechanism derived to simulate natural gas combustion, including NO formation and reburn chemistry. This method is a widely recognized good mechanism for natural gas in much research, e.g., [38,39,40]. The pressure range for GRI Mech 3.0 is wide from 0.1 to 10 atm. The main gas for San Diego [26] is hydrocarbons. San Diego can use the minimum number of reactions and species to explain the phenomenon to increase accuracy compared with GRI 3.0. USC Mech II is a detailed kinetic mechanism specialized for a large number of combustion processes from C0 to C4 with 111 species in 784 reversible reactions [28]. The main gas used for USC Mech II is syngas which is consistent with H_2_ and CO in the high-temperature oxidation process [41]. The Konnov mechanism is very exact chemistry for low temperature and radical effects. The current version of the mechanism (Release 0.5) consists of 1200 reactions among 127 species [27].

## 3. Results and Discussion

### 3.1. Effect of CO_2_ Addition on Laminar Flame Velocity

**Figure 5:**
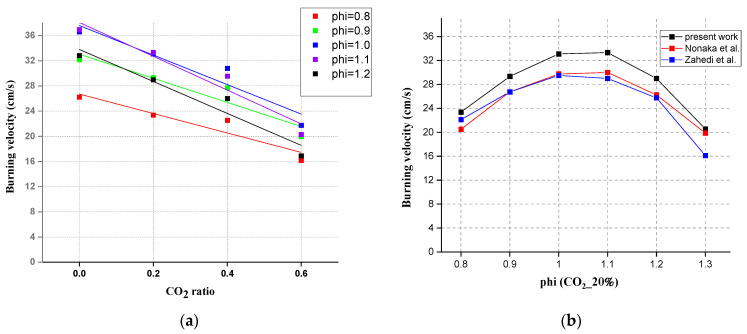
(**a**) Measured data: laminar burning velocity with the addition of CO_2_, (**b**) adiabatic laminar burning velocities with literature data [3,42].

The variation principle of laminar burning velocity with the addition of CO_2_ is shown in Figure 5 below. The laminar burning velocity all show a linear downward trend under different equivalence ratios. When the addition ratio of CO_2_ is from 40 to 60%, the decreasing rate of S_L_ becomes larger. When the addition ratio of CO_2_ is from 20 to 40%, the decreasing rate of S_L_ becomes smaller. Figure 5b shows a comparison with the data from the literature.

### 3.2. Comparison between Experimental and Simulation Results Using Different Mechanisms

There were four mechanisms chosen in this study, which are GRI-Mech 3.0 [25], San Diego [26], Konnov [27], and USC Mech II [28]. Because they are the common mechanisms for gas simulations, like H_2_, CH_4_ etc. For the CO_2_/CH_4_ mixtures, the compositions investigated are: 0/100, 20/80, 40/60, and 60/40 %/% (all by volume fraction). The range of equivalent ratio Φ is from 0.7 to 1.3. The pressure is 1 atm and the temperature is 298 K. Both burning velocities and CO and NO emissions were reported. The results for CO emissions and NO emissions were taken at a downstream position of 20 cm, the latter as measured in the accompanying experiments.

**Figure 6:**
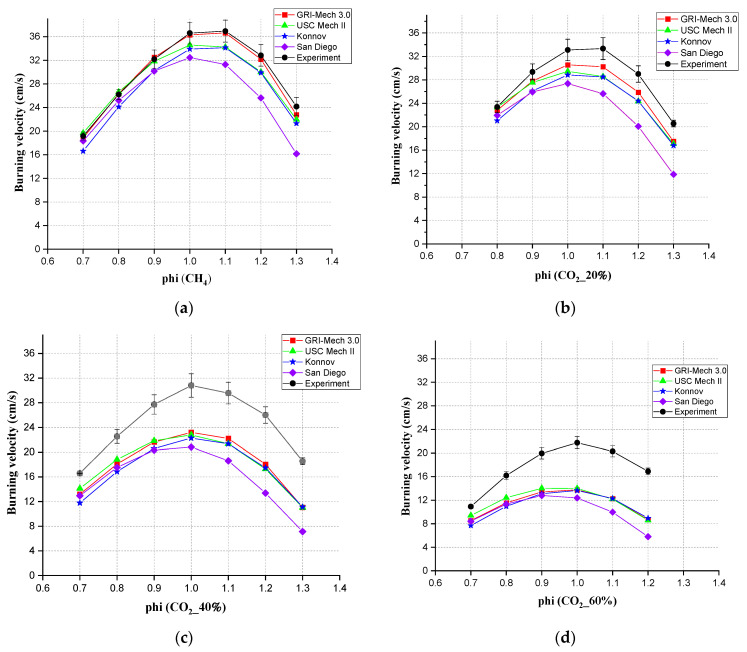
Comparison between simulation values (different simulation mechanisms) and experimental value for different mixtures, (**a**) CH_4_, (**b**) 20% CO_2_, (**c**) 40% CO_2_, (**d**) 60% CO_2_.

From Figure 6, it can be observed that all mechanisms involved showed the same tendency. The simulation results were all lower than the experimental results. GRI Mech 3.0 showed the best agreement when the CO_2_ content was below 20%. When CO_2_ accounted lower than 20%, the ranking of accuracy among all four mechanisms was GRI-Mech 3.0 > USC Mech II > Konnov > San Diego. When CO_2_ accounted for 40 and 60%, the ranking of accuracy interchanged between the first two. All in all, GRI-Mech 3.0 was the best choice for S_L_ simulation of mixed gas in Chem1D when CO_2_ accounted for lower than 20%. USC Mech II was a good mechanism when the mixed gas had a higher concentration (>40%) of CO_2_.

When the gas was CH_4_ and when the CO_2_ accounted for 20%, the highest values of S_L_ appeared at Φ = 1.1 in the experiments. In other proportions of CO_2_ in this experiment, the highest values of S_L_ were found at Φ = 1.0; therefore, the highest S_L_ mainly appeared in the interval from 1 to 1.1 in the experiment. But the Chem1D simulation results showed the highest S_L_ at Φ = 1.1 for CH_4_ for GRI 3.0 and Konnov. For other ratios of mixed gases, simulations with different mechanisms did not give a synchronous prediction for the peak value of S_L_. The peak value of S_L_ for other mixed gases all appeared at Φ = 1.0 from Chem1D simulation results, so the experiments presented the change of S_L_ in a more trustworthy manner.

### 3.3. Comparison of Experimental and Simulated Values of Different Concentrations of CO_2_

**Figure 7:**
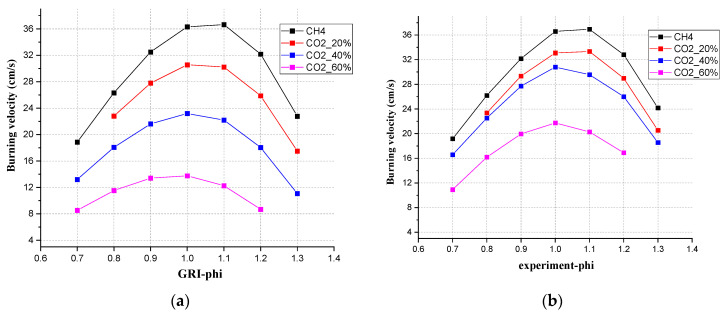
The burning velocity for different CO_2_ compositions (**a**) from the experiment, and (**b**) from simulations with GRI 3.0.

From both simulation and experiment, the changing trends of S_L_ showed a consistent pattern. In Figure 7, the behavior was shown for different mixtures in experiments and for simulations with GRI 3.0. When the range of Φ was between 0.7 and 1.0, as Φ increased, S_L_ showed an increasing trend as well. When the range of Φ was between 1.0 and 1.3, as Φ increased, S_L_ showed a decreasing trend. The authors used the mechanism of GRI Mech 3.0 to show how the S_L_ changed with the concentration of CO_2_ in the simulation. As the concentration of CO_2_ increased every 10%, under the same Φ, the S_L_ also showed a corresponding regular decrease. From the experimental results, under the same Φ, the S_L_ decreased as well.

### 3.4. The Emission of CO, Simulation and Experimental Results at 20 cm Downstream

**Figure 8:**
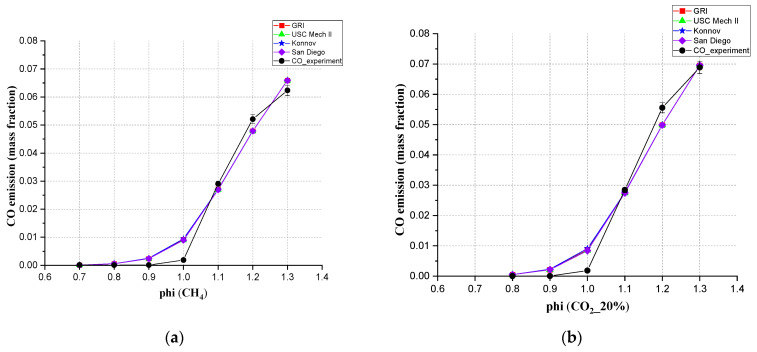

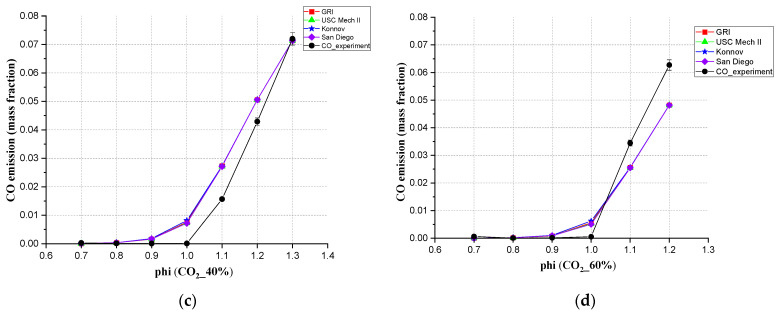
Comparison between simulation values (different simulation mechanisms at 20 cm downstream position) and experimental values for the mixed gas at (**a**) CH_4_, (**b**) 20% CO_2_, (**c**) 40% CO_2_, (**d**) 60% CO_2_.

From Figure 8, for the CO emissions at the location of the measurement probe, the results from the four kinetic mechanisms used in Chem1D showed very little deviation from each other, though a small deviation with experiment can be observed. Under every ratio of CO_2_ content, the CO emissions increase as Φ increases. For the pure CH_4_ and mixed gas with a CO_2_ ratio below 40%, the experimental results showed good consistency with the simulation results. At lean conditions, the CO emissions are almost zero as expected. Then, in rich conditions, when there is insufficient O_2_ for complete combustion, it increased rapidly after Φ = 1.0. After Φ = 1.0, the experimental results showed a higher CO emission than simulation. Like the Figure 8d of CO_2_ = 60%. This is consistent with the theoretical principle. More CO will emerge under the rich CO_2_ situation. The simulation results all showed a continuous acceleration of growth rate from Φ = 1.0 to Φ = 1.3. From the experiment, when CO_2_ accounts for lower than 40%, the increasing slope of CO emission was first steep from Φ = 1.0 to Φ = 1.2, then became slow from Φ = 1.2 to Φ = 1.3.

**Figure 9:**
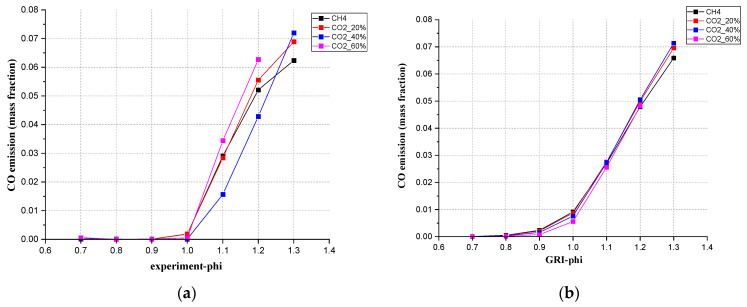

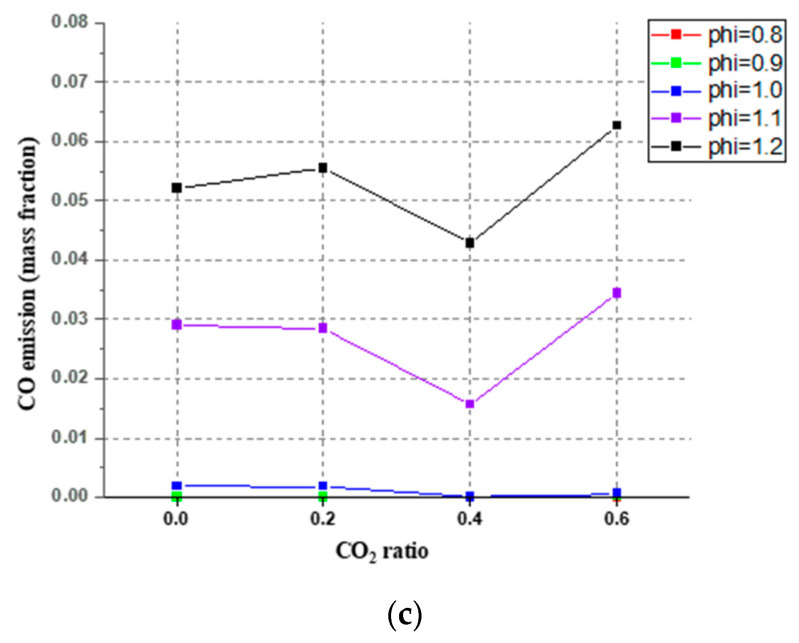
CO emission (**a**) measured, (**b**) simulated with GRI 3.0, (**c**) measured under different CO_2_ ratios.

Figure 9 showed how CO_2_ concentrations affected CO emission from the perspectives of simulation and experiments. From the Chem1D simulation, adding CO_2_ to CH_4_ will produce more exhaust CO emissions when the CO_2_ ratio is between 0–40% in rich conditions. Between 40 to 60%, the CO emission amount will decrease when more CO_2_ is added at these equivalence ratios. From the experiment, adding CO_2_ to CH_4_ from 0 to 20%, the emission of CO had a significant increase. From the simulation, when CO_2_ = 40%, the value of CO emission reached the highest. In the experiment, the highest value of emission appeared at the CO_2_ concentration of 60%.

The conclusion can be drawn here that for the mixed gas, all four mechanisms in Chem1D can be used to predict CO exhaust amount, especially when CO_2_ accounts for less than 40%. These four mechanisms all showed a small error compared with the experiments. In the mixed gas, the proportion of CO_2_ does affect CO emissions. But only when the CO_2_ ratio was less than 20%, compared with the pure CH_4_ gas, the impact was big. When the CO_2_ ratio was more than 20%, the proportion of CO_2_ did not affect CO emissions so much.

### 3.5. The Emission of NO between Simulation (20 cm Downstream Position) and Experimental Results

**Figure 10:**
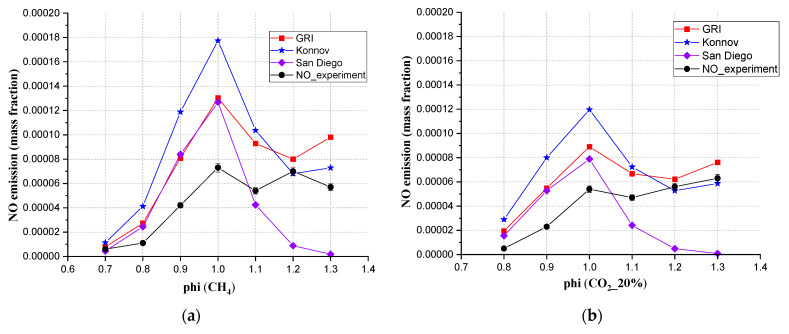

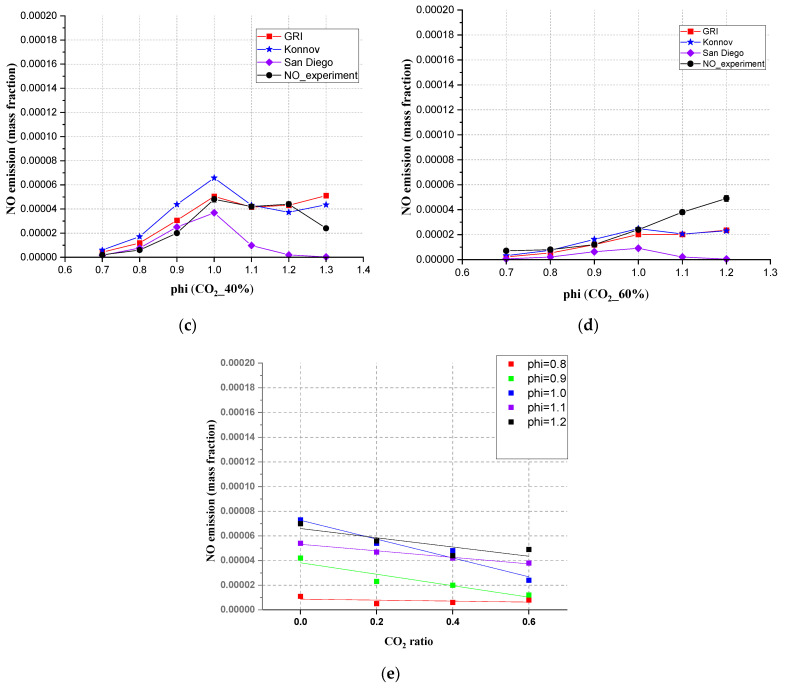
The comparison between simulation values (different simulation mechanisms) and experimental values for the mixed gas under every CO_2_ ratio at 20 cm downstream, (**a**) CH_4_, (**b**) 20% CO_2_, (**c**) 40% CO_2_, (**d**) 60% CO_2_. (**e**) The NO emission measured under different CO_2_ ratios.

The authors used three mechanisms to simulate NO emissions, which were GRI-Mech 3.0, Konnov, and San Diego. The USC Mech II mechanism programming does not involve the calculation of NO. From Figure 10, when the CO_2_ content was 40% and the Φ was between 0.7 to 1.2, the results from simulation (GRI-Mech 3.0) and experiment showed a good agreement. From the experimental results, the NO emission maintained a stable and low level, and showed a linear decreasing relationship as CO_2_ was added. All simulation results were higher than the experimental results. GRI 3.0 showed the smallest differences, therefore, GRI-Mech 3.0 was the best mechanism in this study to simulate NO emissions for mixed gases.

**Figure 11:**
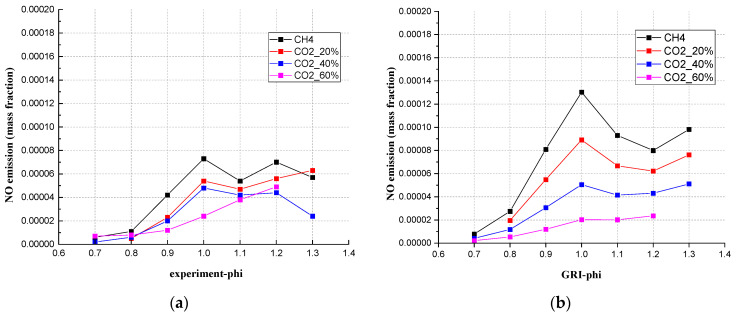
The NO emission of different CO_2_ compositions from (**a**) experiment, (**b**) simulated with GRI 3.0.

From the simulation results of GRI Mech 3.0 in Figure 11b, the NO emission showed a proportional decline with the proportional addition of CO_2_. Of course, as an inert CO_2_ only lowers the temperature and reduces the Zeldovich effect. The experiment results from Figure 11a did not show a similar proportional decline but still showed a reduction tendency. When the equivalence ratio was 1.0, the order of the NO mass fraction was contrary to the concentration of CO_2,_ which means the CO_2_ content affected the NO emission at Φ = 1.0.

### 3.6. Burning Velocities with Different Diffusion Methods

**Figure 12:**
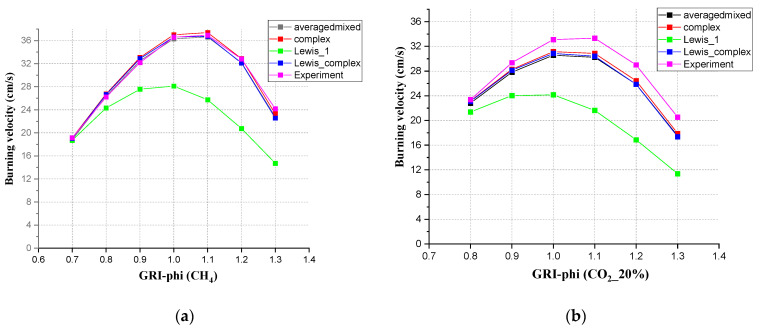
Burning velocities with different diffusion approaches using GRI 3.0 (**a**) for methane, (**b**) for the mixed gas.

Different diffusion approximations were used, complex, mixture averaged, constant Lewis numbers, and unity Lewis numbers. Of course, the complex and mixture averaged approximations showed the best agreement. The authors used the mixture averaged approach for burning velocity, CO emission, and NO emission in this research. The influence of the Lewis number on the numerical simulation results was studied in this chapter. A constant Lewis number approach is often used for analytical considerations. The setting of the Lewis number depends on the problem of concern. If the problem of concern is the thermal effect, the Lewis numbers are reasonable to be 1, but if the focus is on the concentration of light molecules and their flame behavior, detailed complex diffusion must be used. This can explain why the constant Lewis number showed the worst agreement compared with other diffusion approximations. From Figure 12, for CH_4_, the curves from complex, mixture averaged, and unity Lewis numbers showed a very small deviation compared with the experiment results. For the mixture gas, like Figure 12b, when CO_2_ accounted for 20%, there was a bigger deviation between the experimental results and simulation.

## 4. Conclusions

The experimental and numerical study of the laminar burning velocity and pollutant emissions of the mixture gas of methane and carbon dioxide were studied in this research. Compared with previous studies, a wider range of experimental conditions is realized in this paper: CO_2_ dilution level up to 60% and equivalence ratio of 0.7–1.3. Furthermore, this research focused on the emissions of CO and NO after premixed combustion, which filled the gaps in other people’s research. Depending on the results got in this research, the following conclusions can be given:

1. For laminar burning velocity simulation, four chemical kinetic mechanisms, GRI-Mech 3.0, San Diego, Konnov, and USC Mech II, all showed the same tendency compared with the experimental results. The simulation results were all lower than the experimental results. Consistent with the conclusion of Nonaka and Pereira [3], GRI Mech 3.0 showed the best agreement when the CO_2_ content was 20%. USC Mech II showed the best consistency when the CO_2_ content was between 40% and 60%. Agreement was limited when there were CO_2_ additions. The burning velocity showed a liner decrease while adding CO_2._
2. For the CO emission, for the mixed gas, all four mechanisms in Chem1D can be used to predict CO exhaust amount. These four mechanisms all showed a small error compared with the experiments. When CO_2_ content was higher than 40%, the deviation between simulation and experiment became bigger. In the mixed gas, the proportion of CO_2_ did affect CO emissions, making the CO emissions decrease first and then increase.
3. For the NO emission, when the CO_2_ content was 40%, compared with the experimental results, the Chem1D can predict the mixed gas NO emission interval range. The NO emissions showed a linear relationship with the addition of CO_2_. In rich conditions, no matter how much CO_2_ accounted for, the NO emissions were all below 0.0001 mass fraction.
4. All in all, numerical simulation is a good way to predict burning velocities and CO emissions for 1D adiabatic flames. GRI Mech 3.0 is the best kinetic mechanism for this with the current mixtures. Adding CO_2_ to CH_4_ decreases the burning velocity but also decreases NO emissions and does not produce more CO emissions.
5. Different diffusion approximations were used, complex, mixture averaged, constant Lewis numbers, and unity Lewis numbers. Of course, the complex and mixture averaged approximations showed the best agreement. For CH_4_, the curves from complex, mixture averaged, and unity Lewis numbers showed a very small deviation compared with the experiment results. Different burning velocity approximations cause different burning rates estimations and, therefore, different CO and NOx emission rates. The exact impact should be investigated in an upcoming survey. For now, we can say that for truth-finding, complex diffusion is advised for use.
6. The authors found the mixed ratio for CO_2_ as 40% was a good balance between S_L_ and pollutant emissions. For S_L_, the curve from CO_2_ = 40% did not show a big difference compared with CH_4_ but had a much lower CO and NO emission specifically at the point of Φ = 1.0. This is due to the inert dilution that lowers the product’s temperature and, therefore, the thermal (Zeldovich) mechanism; thus, adding CO_2_ into CH_4_ is a good NO_x_ removal method due to its low cost. Good advice can be provided here for industrial utilization by using 40% CO_2_ of the natural gas, which can reduce the pollutant emission without drastically reducing the burning velocity. More simulation work to research the ignition delay time, chemical and thermal effects on burning velocity will be undertaken in the future.

## References

[1] Rasi S., Veijanen A., Rintala J. (2007). Trace Compounds of Biogas from Different Biogas Production Plants. Energy.

[2] Jönsson O., Polman E., Jensen J.K., Eklund R., Schyl H., Ivarsson S. (2003). Sustainable Gas Enters the European Gas Distribution System.

[3] Nonaka H.O.B., Pereira F.M. (2016). Experimental and Numerical Study of CO_2_ Content Effects on the Laminar Burning Velocity of Biogas. Fuel.

[4] Goswami M., Van Griensven J.G.H., Bastiaans R.J.M., Konnov A.A., De Goey L.P.H. (2015). Experimental and Modeling Study of the Effect of Elevated Pressure on Lean High-Hydrogen Syngas Flames. Proc. Combust. Inst..

[5] Andrews G.E., Bradley D. (1972). Determination of Burning Velocities: A Critical Review. Combust. Flame.

[6] Zhao P., Yuan W., Sun H., Li Y., Kelley A.P., Zheng X., Law C.K. (2015). Laminar Flame Speeds, Counterflow Ignition, and Kinetic Modeling of the Butene Isomers. Proc. Combust. Inst..

[7] de GOEY L.P.H., van MAAREN A., QUAX R.M. (1993). Stabilization of Adiabatic Premixed Laminar Flames on a Flat Flame Burner. Combust. Sci. Technol..

[8] Rallis C.J., Garforth A.M. (1980). The Determination of Laminar Burning Velocity. Prog. Energy Combust. Sci..

[9] Cohe C., Chauveau C., Gökalp I., Kurtuluş D.F. (2009). CO_2_ Addition and Pressure Effects on Laminar and Turbulent Lean Premixed CH_4_ Air Flames. Proc. Combust. Inst..

[10] Galmiche B., Halter F., Foucher F., Dagaut P. (2011). Effects of Dilution on Laminar Burning Velocity of Premixed Methane/Air Flames. Energy Fuels.

[11] Halter F., Foucher F., Landry L., Mounaïm-Rousselle C. (2009). Effect of Dilution by Nitrogen and/or Carbon Dioxide on Methane and Iso-Octane Air Flames. Combust. Sci. Technol..

[12] Xie Y., Wang J., Zhang M., Gong J., Jin W., Huang Z. (2013). Experimental and Numerical Study on Laminar Flame Characteristics of Methane Oxy-Fuel Mixtures Highly Diluted with CO_2_. Energy Fuels.

[13] Chen Z., Tang C., Fu J., Jiang X., Li Q., Wei L., Huang Z. (2012). Experimental and Numerical Investigation on Diluted DME Flames: Thermal and Chemical Kinetic Effects on Laminar Flame Speeds. Fuel.

[14] Qiao L., Dahm W., Faeth G., Oran E. Burning Velocities and Flammability Limits of Premixed Methane/Air/Diluent Flames in Microgravity. Proceedings of the 46th AIAA Aerospace Sciences Meeting and Exhibit.

[15] Van Maaren A., Thung D.S., DE GOEY L.R.H. (1994). Measurement of Flame Temperature and Adiabatic Burning Velocity of Methane/Air Mixtures. Combust. Sci. Technol..

[16] Hermanns R.T.E., Kortendijk J.A., Bastiaans R.J.M., De Goey L.P.H. (2007). Laminar Burning Velocities of Methane-Hydrogen-Air Mixtures. Ph.D. Thesis.

[17] Dyakov I.V., Konnov A.A., Ruyck J.D., Bosschaart K.J., Brock E.C.M., De Goey L.P.H. (2001). Measurement of Adiabatic Burning Velocity in Methane-Oxygen-Nitrogen Mixtures. Combust. Sci. Technol..

[18] Konnov A.A., Dyakov I.V. (2004). Measurement of Propagation Speeds in Adiabatic Flat and Cellular Premixed Flames of C_2_H_6_+ O_2_+ CO_2_. Combust. Flame.

[19] Konnov A.A., Dyakov I.V. (2005). Measurement of Propagation Speeds in Adiabatic Cellular Premixed Flames of CH_4_+ O_2_+ CO_2_. Exp. Therm. Fluid Sci..

[20] Coppens F.H.V., De Ruyck J., Konnov A.A. (2007). Effects of Hydrogen Enrichment on Adiabatic Burning Velocity and NO Formation in Methane+ Air Flames. Exp. Therm. Fluid Sci..

[21] Clarke A., Stone R., Beckwith P. (1995). Measuring the Laminar Burning Velocity of Methane/Diluent/Air Mixtures within a Constant-Volume Combustion Bomb in a Micro-Gravity Environment. Fuel Energy Abstr..

[22] Skalska K., Miller J.S., Ledakowicz S. (2010). Trends in NOx Abatement: A Review. Sci. Total Environ..

[23] Abbasfard H., Hashemi S.H., Rahimpour M.R., Jokar S.M., Ghader S. (2013). Reducing NO x Emissions from a Nitric Acid Plant of Domestic Petrochemical Complex: Enhanced Conversion in Conventional Radial-Flow Reactor of Selective Catalytic Reduction Process. Environ. Technol..

[24] Somers L.B. (1994). The Simulation of Flat Flames with Detailed and Reduced Chemical Models. Ph.D. Thesis.

[25] Smith G.P., Golden D.M., Frenklach M., Moriarty N.W., Eiteneer B., Goldenberg M., Bowman C.T., Hanson R.K., Song S., Gardiner W. (1999). Gri-Mech 3.0.

[26] William F.A., Kalyanasundaram S., Cattolica R.J. (2012). The San Diego Mechanism—Chemical-Kinetic Mechanisms for Combustion Applications.

[27] Konnov A. (1998). Detailed Reaction Mechanism for Small Hydrocarbons Combustion, Release 0.4. http://homepages.vub.ac.be/~akonnov/.

[28] Wang H., You X., Joshi A.V., Davis S.G., Laskin A., Egolfopoulos F., Law C.K. (2007). USC Mech Version II. High-Temperature Combustion Reaction Model of H_2_/CO/C1-C4 Compounds.

[29] Hinton N., Stone R. (2014). Laminar Burning Velocity Measurements of Methane and Carbon Dioxide Mixtures (Biogas) over Wide Ranging Temperatures and Pressures. Fuel.

[30] Powling J. (1949). A New Burner Method for the Determination of Low Burning Velocities and Limits of Inflammability. Fuel.

[31] Bosschaart K.J., De Goey L.P.H. (2003). Detailed Analysis of the Heat Flux Method for Measuring Burning Velocities. Combust. Flame.

[32] Raida M.B., Hoetmer G.J., Konnov A.A., van Oijen J.A., de Goey L.P.H. (2021). Laminar Burning Velocity Measurements of Ethanol+air and Methanol+air Flames at Atmospheric and Elevated Pressures Using a New Heat Flux Setup. Combust. Flame.

[33] Alekseev V.A., Naucler J.D., Christensen M., Nilsson E.J.K., Volkov E.N., de Goey L.P.H., Konnov A.A. (2016). Experimental Uncertainties of the Heat Flux Method for Measuring Burning Velocities. Combust. Sci. Technol..

[34] Burcat A., Ruscic B. (2005). Third Millenium Ideal Gas and Condensed Phase Thermochemical Database for Combustion (with Update from Active Thermochemical Tables).

[35] Li J., Zhao Z., Kazakov A., Chaos M., Dryer F.L., Scire J.J. (2007). A Comprehensive Kinetic Mechanism for CO, CH_2_O, and CH_3_OH Combustion. Int. J. Chem. Kinet..

[36] Burke M.P., Chaos M., Ju Y., Dryer F.L., Klippenstein S.J. (2012). Comprehensive H_2_/O_2_ Kinetic Model for High-Pressure Combustion. Int. J. Chem. Kinet..

[37] Kéromnès A., Metcalfe W.K., Heufer K.A., Donohoe N., Das A.K., Sung C.-J., Herzler J., Naumann C., Griebel P., Mathieu O. (2013). An Experimental and Detailed Chemical Kinetic Modeling Study of Hydrogen and Syngas Mixture Oxidation at Elevated Pressures. Combust. Flame.

[38] Bosschaart K.J., Versluis M., Knikker R., Vandermeer T.H., Schreel K., De Goey L.P.H., Van Steenhoven A.A. (2001). The Heat Flux Method for Producing Burner Stabilized Adiabatic Flames: An Evaluation with CARS Thermometry. Combust. Sci. Technol..

[39] Hermanns R.T.E., Konnov A.A., Bastiaans R.J.M., de Goey L.P.H., Lucka K., Köhne H. (2010). Effects of Temperature and Composition on the Laminar Burning Velocity of CH_4_+H_2_+O_2_+N_2_ Flames. Fuel.

[40] Goswami M., Derks S.C.R., Coumans K., Slikker W.J., de Andrade Oliveira M.H., Bastiaans R.J.M., Luijten C.C.M., de Goey L.P.H., Konnov A.A. (2013). The Effect of Elevated Pressures on the Laminar Burning Velocity of Methane+air Mixtures. Combust. Flame.

[41] He Y., Wang Z., Weng W., Zhu Y., Zhou J., Cen K. (2014). Effects of CO Content on Laminar Burning Velocity of Typical Syngas by Heat Flux Method and Kinetic Modeling. Int. J. Hydrog. Energy.

[42] Zahedi P., Yousefi K. (2014). Effects of Pressure and Carbon Dioxide, Hydrogen and Nitrogen Concentration on Laminar Burning Velocities and NO Formation of Methane-Air Mixtures. J. Mech. Sci. Technol..

